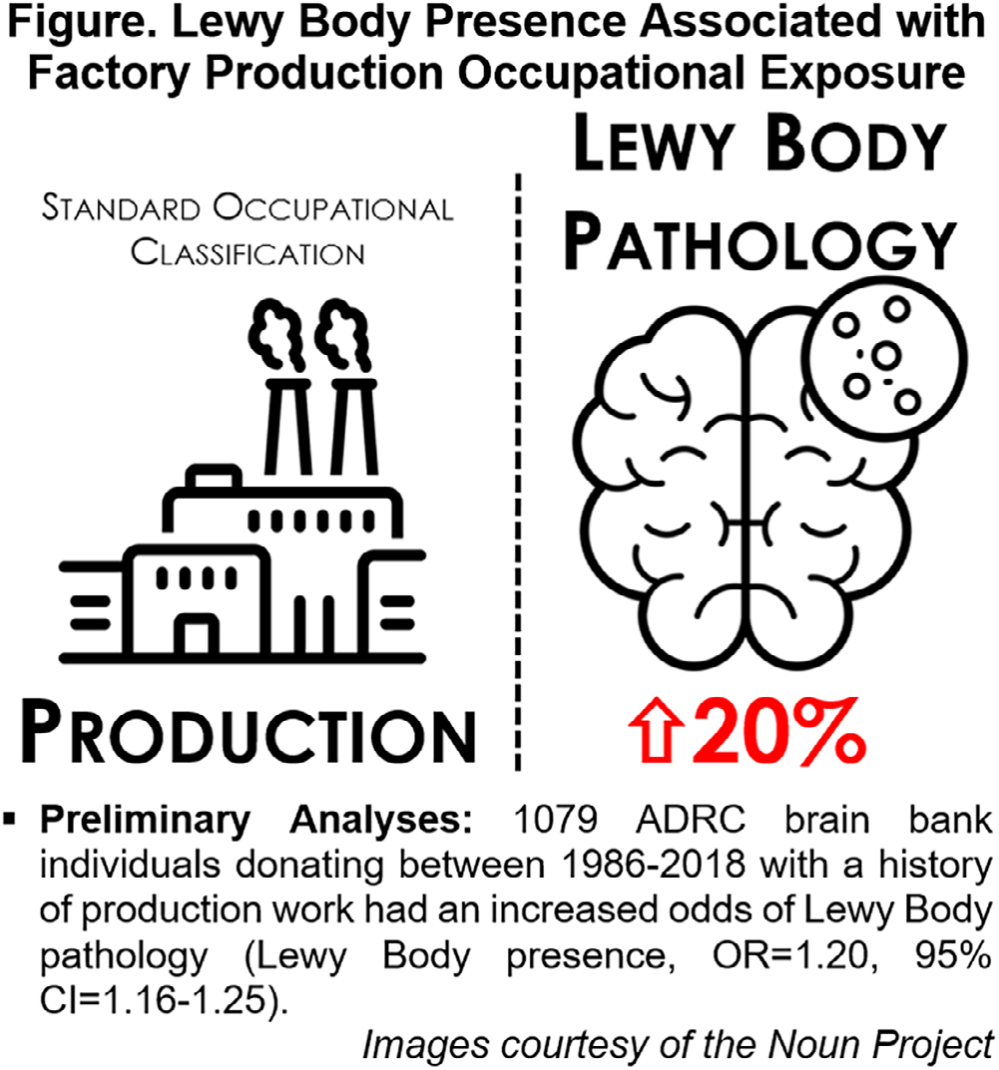# Association of Lifecourse Factory Production Work and Lewy Body Neuropathology

**DOI:** 10.1002/alz70861_108660

**Published:** 2025-12-23

**Authors:** W. Ryan Powell, Eleanna M. Melcher, Lauren Etter, Rachel C. Otte, Sarah Lim, Aly Pfaff, Amanda DeWitt, Adrienne R.S. Lee, Fred B. Ketchum, Shahriar Salamat, Megan L. Zuelsdorff, Stephen A. Goutman, Kelly M. Bakulski, Robert A. Rissman, Barbara B. Bendlin, Amy J.H. Kind

**Affiliations:** ^1^ Center for Health Disparities Research, University of Wisconsin School of Medicine and Public Health, Madison, WI USA; ^2^ Department of Medicine, Geriatrics Division, University of Wisconsin School of Medicine and Public Health, Madison, WI USA; ^3^ Department of Population Health Sciences, University of Wisconsin School of Medicine and Public Health, Madison, WI USA; ^4^ Department of Medical Physics, University of Wisconsin School of Medicine and Public Health, Madison, WI USA; ^5^ Department of Neurology, University of Wisconsin‐Madison School of Medicine and Public Health, Madison, WI USA; ^6^ Department of Neurological Surgery, University of Wisconsin School of Medicine and Public Health, Madison, WI USA; ^7^ Department of Pathology and Laboratory Medicine, University of Wisconsin School of Medicine and Public Health, Madison, WI USA; ^8^ Wisconsin Alzheimer's Disease Research Center, University of Wisconsin School of Medicine and Public Health, Madison, WI USA; ^9^ University of Wisconsin School of Nursing, Madison, WI USA; ^10^ Department of Neurology, University of Michigan Medical School, Ann Arbor, MI USA; ^11^ University of Michigan School of Public Health, Ann Arbor, MI USA; ^12^ Michigan Alzheimer's Disease Research Center, University of Michigan Medical School, Ann Arbor, MI USA; ^13^ Keck School of Medicine of the University of Southern California, Los Angeles, CA USA; ^14^ Wisconsin Alzheimer's Institute, University of Wisconsin School of Medicine and Public Health, Madison, WI USA

## Abstract

**Background:**

Work as a determinant of health and related disparities may be an important source of occupational exposures, yet its role in influencing the biological underpinnings of Alzheimer’s disease and related dementias (ADRD), including Lewy Body dementia, is limited. Identifying high risk occupational exposures is a critical step in understanding the roles that work has in explaining ADRD, and could lead to effective design of disease prevention and intervention strategies for the most efficient allocation of resources.

**Method:**

The sample included Individuals donating to two Alzheimer's Disease Research Center (ADRC) brain banks from 1986 to 2018 with Lewy body pathology assessed by a neuropathologist (n = 1079). Donor occupational histories derived from archival public records went through a multi‐step process to assign a 6‐digit occupation Standardized Occupation Code (SOC) for each occupation using established protocols. Using generalized logistic regression, we modeled the presence of Lewy bodies association with having ever worked in SOC category “Production Occupations” (SOC=51‐XXXX) adjusting for age, sex, and death year with ADRC‐site clustered standard errors.

**Result:**

Lewy Bodies were present in 32% of the total sample. Donors with a history of “Production Occupations” (12.5% of the sample) face a 20% increased adjusted odds of having Lewy Bodies (Figure OR=1.20, 95% CI=1.16‐1.25) relative to donors without a history of "Production Occupations".

**Conclusion:**

This is the first study, to our knowledge, finding increased levels of verified Lewy Body neuropathology in those with “Production Occupations”—i.e., donors who reported working as factory workers, foremans, assemblers, among other occupations in their work histories. Additional precision categorizing industrial production and manufacturing exposure in their occupational histories is needed to identify specific drivers behind this association, which may include the direct exposure to environmental hazards including heavy metal exposure.